# The longer-term effects of IVF on offspring from childhood to adolescence

**DOI:** 10.3389/frph.2022.1045762

**Published:** 2022-12-08

**Authors:** R. J. Hart, L. A. Wijs

**Affiliations:** ^1^ Division of Obstetrics and Gynaecology, University of Western Australia, Perth, WA, Australia; ^2^ Fertility Specialists of Western Australia, Bethesda Hospital, Claremont, WA, Australia; ^3^ City Fertility Clinic, Brisbane, QLD, Australia

**Keywords:** IVF, long-term, adolescent, outcome, offspring, health, children, assisted reproductive technologies (ART)

## Abstract

It is well established that there are increased pregnancy-related complications for a woman who conceives through assisted reproductive treatment (ART). Furthermore, it is known that the risk to the child born is greater, believed to be related to prematurity and growth restriction. Studies have also reported epigenetic changes in the DNA of offspring conceived through ART. In addition, it is believed that they have a greater risk of congenital malformations, although some of these risks may relate to underlying infertility, rather than the ART treatment *per se*. As a result, it may be expected that there is a greater risk to the longer-term health of the child who is born from ART; however, evidence about the long-term health of children conceived through ART is reassuring. Even though, it is recognised that many of the studies in this field come with limitations. Low numbers of participants is one of the major limitations, which makes subgroup analyses for diverse types of ART, or diverse types of infertility, not feasible. Furthermore, studies are often limited by short follow-up periods because of the difficulty and costs involved in longitudinal study designs. In addition, the rapid changes over time in ART limit the generalisability and significance of long-term findings. Well-designed studies investigating the long-term health outcomes of ART-conceived offspring and the potential influences of various aspects of the ART procedure, as well as studies of the potential underlying epigenetic mechanisms, are imperative. Furthermore, conclusions from childhood hospitalisation data from the United Kingdom, the long-term follow-up and quality of life study from researchers in Melbourne, and the data published from the Western Australian Growing Up Healthy Study will go a long way to help reassure current and prospective parents who may require ART to conceive.

## Introduction

It is believed that over 10 million children have been born subsequent to assisted reproductive technologies (ART) globally (1), and approximately 2.5 million ART cycles are performed annually, resulting in approximately 500,000 babies born each year (1). However, despite these statistics, it is disappointing that we are not able to provide prospective ART parents with clarity as to the longer-term health outcome of the children born subsequent to ART (2). Although the obstetric outcomes for women who conceive with ART and the short-term outcomes of their children born from ART have been extensively studied, they suggest some potential early life predictors of disease in later life (2–4).

It has been well established over the short term that ART pregnancies are associated with a higher prevalence of pregnancy and perinatal complications (5–8). Consequently, it may be hypothesised that ART treatment may lead to adverse long-term health outcomes for the children born, along the lines of the development of adult health and disease hypothesis (9). Hence, it is essential to study the health outcomes of ART-conceived offspring beyond the short horizon of the perinatal and childhood periods. Presented is a narrative review that focuses on the literature reporting on the health outcomes of adolescents conceived from ART. It is essential that we are able to provide prospective parents with reliable information as to the potential health outcomes and health trajectory into adulthood for their future children. Even though, it is acknowledged that over time the technologies employed in ART have evolved, with the continued shift from cleavage stage to blastocyst embryo culture, and from the slow-freezing of embryos to vitrification for cryopreservation, and with the introduction of pre-implantation genetic testing of embryos, it is recognised that outcomes today will be potentially different to outcomes in the future.

As stated, despite the best intentions of the researchers, the nature of the technology involved in ART results in the fact that many follow-up studies of children born from ART have understandable limitations. Many ART studies include retrospectively collected data, have difficulty establishing a representative comparator group, and relate to in-vitro fertilisation (IVF) practices that are obsolete or have evolved. Consequently, to date, studies show conflicting results regarding the longer-term health outcomes of the offspring, with some reports of reassuring findings. However, others report evidence of differences in cardiometabolic and cardiovascular parameters, endocrine parameters and growth, psychological and cognitive health, childhood cancers, immunological disorders such as asthma and allergies, and reproductive health (3). For example, various childhood studies have demonstrated an increase in systolic and diastolic blood pressure, adiposity, vascular dysfunction, as well as unfavourable lipid and glucose profiles (10). However, more recent studies report no differences, or indeed favourable cardiometabolic outcomes, for ART-conceived offspring (11–14). However, studies performed on adolescents are scarce. Of particular relevance, several studies have reported differences between ART-conceived and naturally conceived offspring with regard to mental health outcomes, reporting a potentially higher prevalence of depression, autism spectrum disorder, attention-deficit disorder, and behavioural differences (15–17). Furthermore, there are conflicting reports as to whether asthma and allergies are more prevalent in the offspring of ART (18, 19).

In our reviews of the longer-term health outcomes of ART-conceived offspring (2, 20), acknowledging the limitations of the studies with respect to their small sample sizes and challenges relating to adequate comparator groups, we reported a potentially higher prevalence of thyroid disorders, increased early life growth velocity, raised blood pressure, elevated fasting glucose, higher total body fat composition, an advancement of bone age, a higher prevalence of early adulthood clinical depression, attention-deficit hyperactivity disorder (ADHD), and binge drinking (2, 20). Differences in study findings to date are potentially explained by differences in study designs and covariate adjustment. In addition, given the rapid changes in ART practice, results from more recent “younger” cohorts may differ from those from earlier cohorts. In a review by Berntsen et al., it is suggested that improved and milder stimulation protocols, and possibly changes to freezing technology employed, may lead to fewer epigenetic modifications in the embryo (10). It has further been suggested that the epigenetic alterations reported in ART-conceived newborns may be mitigated by adolescence; however, further studies are required (21, 22). In view of these potential health concerns for ART-conceived offspring and given the lack of studies beyond childhood, one author (RJH) established the Growing Up Healthy Study (GUHS) (23). The purpose of the project was to study the health outcomes of adolescents and young adults born from ART in Western Australia prior to 2001 and to compare them to an established birth cohort, the Raine cohort (24), which has been demonstrated to be representative of the wider Western Australian population, by replicating the same detailed adolescent clinical assessments. This paper includes an overview of some of the findings from this study.

## Potential mechanisms of adverse health outcomes

It is perhaps not surprising that there are concerns about the health of the offspring from ART as pregnancies resulting from ART have a higher prevalence of obstetric complications, such as hypertensive disorders, pre-eclampsia, gestational diabetes, placental problems, premature delivery, and a greater requirement for induction of labour and caesarean sections (5–8), even after controlling for multiple gestations (25). Indeed, the short-term health outcomes of offspring conceived after ART have been extensively studied (26). It is known that ART-conceived offspring are at an increased risk of preterm birth (27, 28), low birth weight (27–29), fetal growth restriction (30), congenital malformations (5, 31), imprinting disorders (32), and neonatal death (5, 31, 33). As a consequence of this, there is some evidence that ART offspring are at an increased risk of neurodevelopmental disorders, specifically cerebral palsy, although evidence is conflicting. For example, Zhu et al. reported an association between ART and cerebral palsy even after controlling for multiplicity and preterm birth (34), although other studies reported that there was no difference in neurodevelopmental delay (35, 36).

A potential mediator of any adverse longer-term health outcome findings is the finding that subfertility itself is associated with an increased risk of several obstetric complications (37) and congenital malformations (30, 38), irrespective of the use of ART, in comparison to pregnancies in women who conceive rapidly, although this is disputed (39). Furthermore, patients who are subfertile may be older or more likely to have comorbidities such as hypertension, diabetes, thyroid disorders, or polycystic ovary syndrome (PCOS), which in their turn might predispose a woman to adverse obstetric and perinatal outcomes, which may have longer-term consequences (40).

Further mediators of any potential adverse long-term health outcomes is that it is believed that intra-cytoplasmic sperm injection (ICSI) is associated with more congenital malformations than IVF (41) and that babies born subsequent to frozen embryo transfer are larger than those after a fresh transfer (42), and different embryo culture media may exert different influences over growth patterns in children (43), potentially suggesting alterations of the epigenetic influences over fetal growth.

Of particular note is that the procedure of ART has been associated with a higher rate of imprinting disorders in the offspring (44–46), as the processes involved in ART during this dynamic period of development may adversely impact gene methylation, leading to epigenetic changes in the embryo that may be the origin of disease later in life (47). Furthermore, potential origins of epigenetic changes within the embryo include the health of the couple undergoing the fertility treatment, in addition to all aspects of the ART: from the stimulation through embryo culture to the freezing of the embryo (48, 49). Epigenetic modification of gene expression may occur through DNA methylation, histone modification, chromatin restructure, and non-coding RNA regulation; however, DNA methylation is the commonest. Reassuringly, an Australian Melbourne-based group reported that the differential DNA methylation patterns in ART infants did not persist into adulthood (22). The same group also reported that some of the childhood and adolescent-associated adverse health outcomes are short-lived and may have resolved by adulthood (12). They reported no differences in the growth, respiratory health, cardiovascular, or metabolic risks of 193 adults who were conceived by IVF when they were compared with 86 naturally conceived adults. In addition, our group performed a comparative analysis of the DNA methylation signatures of ART and naturally conceived adolescent children at 15 years of age (from the Raine cohort) and reported no significant differences in the overall DNA methylation signatures between the groups, irrespective of conception *via* IVF or ICSI, or with a fresh or frozen embryo (23). However, we did not exclude the possibility that alternative epigenetic mechanisms, such as histone modification, imprinting, and non-coding RNA regulation, may be at play (23). In summarising the literature concerning potential epigenetic changes in offspring from ART, Sciorio et al. recently stated, “there is still no conclusive evidence of a strong link between ART and epigenetic modifications as well as increased disease risk in later adult life” (49). Considering all the evidence to date, there is a need for prospective, long-term, and large-scale epidemiological studies, to evaluate the impact of ART on the epigenome of the offspring, as recently summarised by a systematic review and meta-analysis (50).

## The longer-term influences of ART on the offspring

### Hospitalisation

A useful surrogate of health outcomes in a population is the rate of hospitalisation of adolescents born from ART, although it is difficult to control for parental health support seeking behaviour that may influence hospital presentation. A recent follow-up study comparing whole population data of hospital admissions for children born in the United Kingdom from ART treatment (mean age 12.9 years) between 1997 and 2009, in comparison to naturally conceived children, demonstrated generally reassuring findings (4).

ART-conceived children had a slightly higher risk of any hospital admission when compared with their naturally conceived counterparts (hazard ratio 1.08). This increased risk was evident when IVF, with and without ICSI, and fresh and frozen transfers were compared with natural conceptions. Particularly, ART singletons had a greater risk of admission for infectious and parasitic diseases and diseases of the digestive and respiratory systems. The authors related the potential reasons for the increased rate of hospitalisations as possibly due to impaired fetal or childhood growth or potentially social explanations such as increased parental concern, with parents of ART children viewing their offspring as being more vulnerable, leading to a lower threshold for seeking medical support. Other than the substantial size of this study, a further strength was the attempt to include the naturally conceived siblings of the ART offspring in an attempt to control for the social environment. This analysis demonstrated an attenuation of the trend of increased hospital admissions for IVF-conceived children when compared with their naturally conceived siblings, suggesting that the findings could be in part attributed to parental factors, such as the influence of parental subfertility on child health or their increased parental concerns (4).

### Cardiometabolic health

Due to the relatively young age of the individuals conceived through ART, most studies rely on surrogate measures of cardiometabolic health (such as BMI, body fat, blood pressure, serum lipids, glucose tolerance, and inflammatory markers). A recent systematic review and meta-analysis found a minor yet statistically significant increase in both systolic and diastolic blood pressure in IVF/ICSI-conceived offspring compared with their non-ART counterparts. Cardiac diastolic function was suboptimal, and vessel thickness was higher in ART-conceived offspring. Other metabolic factors, such as low-density lipoprotein cholesterol levels, BMI, and fasting insulin, were comparable (51). Although other more recent studies reported no differences in cardiometabolic parameters between ART and non-ART-conceived offspring (11, 13, 14), in our GUHS study 163 ART of offspring at 17 years of age replicated the assessments previously completed by the 1,457 participants in the Raine study at a similar age (52). The ART offspring females had a lower BMI, thinner skinfolds, and less subcutaneous fat, than the Raine participants, whereas males were not significantly different. The ART offspring had a narrower waist circumference than their naturally conceived counterparts, with no differences in their serum metabolic indices, homeostatic model assessment for insulin resistance, the presence of fatty liver, blood pressure, or heart rate than their naturally conceived counterparts. Although, the pulse wave velocity was lower in ART-conceived males than their naturally conceived counterparts, providing reassuring data in cardiometabolic measures for boys and girls in adolescence (52). All data were adjusted for being a multiple gestation, gestational age at delivery, birth weight Z score, smoking, high alcohol intake, recent oral contraceptive use, and parental cardiovascular status.

In view of the fact that ICSI has been more widely embraced over the last 25 years, it is essential to monitor the longer-term follow-up of individuals conceived from ICSI, although perhaps more challenging to do so as it was less frequently adopted until recently. For example, in the GUHS, only 12% of adolescents studied were conceived *via* ICSI (23). However, despite this, the Melbourne group, using the Raine cohort as a comparator, studied a population of ICSI-conceived young men (53). They reported some metabolic differences in 121 ICSI-conceived men aged 18–24 years when compared with 688 spontaneously conceived young men, and 74 men conceived *via* IVF. The differences included higher resting diastolic blood pressure and homeostasis model assessment for insulin resistance scores, although the metabolic parameters of ICSI- and IVF-conceived singleton men were more comparable (53).

### Testicular function

Although not the main focus of this adolescent review, the same Melbourne group studied the testicular function of young men aged 18–25 years born resulting from IVF and ICSI and compared them with young men from the Raine cohort and demonstrated generally reassuring findings (54). They demonstrated that men conceived with IVF/ICSI had similar sperm concentration, total sperm count, and total motile count to spontaneously conceived men; however, there were subtle significant differences in progressive motility, abnormal morphology, gonadotrophins, and serum testosterone concentrations (54).

### Respiratory health and allergies

Contrasting data exist in the literature with respect to the prevalence of asthma in children born from ART, and the data may be confounded, or the outcomes mediated, by the prevalence of maternal asthma, smoking, neonatal morbidity, preterm birth, and low birth weight. Two systematic reviews have reported a mild increased risk of asthma in ART-conceived offspring in comparison to their naturally conceived counterparts, with studies on allergies being limited and less conclusive (18, 19).

At 14 years of age, 152 participants in the GUHS undertook detailed respiratory questionnaires and spirometry, a methacholine challenge test to attempt to evoke a bronchial response, skin prick tests for allergy, and questionnaires for food allergies (55). No differences were detected in the prevalence of current asthma; indeed, the measured lung volumes were larger in the ART adolescents, and bronchial hyper-responsiveness was less prevalent in ART offspring. Although, allergic rhinoconjunctivitis (hay fever) rates were higher in the ART cohort, food allergies were more prevalent, and more adolescents had a positive skin prick test, than the 1,845 naturally conceived Raine Study participants who underwent assessment (55). Whether these differences noted relate to altered immunity, perhaps with epigenetic origins, or differences in parenting controlling early life environmental exposures, will require further study. Respiratory analyses were adjusted for being a multiple birth, a caesarean birth, sex, gestational age at delivery, the birth weight Z score, whether the primary caregiver was a smoker, and height.

### Prevalence of thyroid disorder

Previous studies of thyroid function between ART and non-ART-conceived offspring reported differences in thyroid function (56, 57). In 2009, Sakka et al. conducted a prospective cohort study on thyroid function in 106 IVF-conceived children and 68 randomly selected age- and sex-matched controls conceived without ART, reporting a higher incidence of subclinical hypothyroidism (56).

The thyroid function of 181 offspring of ART within the GUHS was compared with that of 1,359 naturally conceived children in the Raine Study at ages 14 and 20 (58). The mean concentrations of thyroid stimulating hormone (TSH) were similar between the two groups, while free triiodothyronine (fT3) concentration was lower and the mean free thyroxine (fT4) was higher, but both were increased at 20 years of age in comparison to the naturally conceived comparison group, although all measures were within the normal range. Furthermore, the prevalence of thyroid autoimmunity did not differ between cohorts at both ages. There were no differences in the prevalence of subclinical and overt hypo- and hyperthyroidism between the two groups, and thyroid function did not differ between the offspring of fresh and frozen embryo transfers. These findings appear reassuring, although they require further investigation in adulthood (58).

### Behavioural and mental health outcomes

There have been many studies of the behaviour and mental health outcomes of offspring of ART; however, the evidence is inconclusive, and most have focussed on childhood years. Most studies of the emotional wellbeing of offspring conceived after ART are reassuring; however, some studies show evidence of a higher prevalence of mental health disorders, particularly depression (20). In a prospective study of parent-reported outcomes, there were no differences reported in internalising (anxiety and depression) or externalising (delinquent or aggressive) behaviour, or in their child's social development between children conceived subsequent to ART and their naturally conceived counterparts (59).

A few studies have reported on the association between ADHD and ART and demonstrated inconclusive and inconsistent results (17, 60–63). Similarly, studies of any association of ART with autism spectrum disorder in the offspring have generally focussed on younger children and are inconclusive (62, 64, 65).

In the GUHS, the behaviour and mental health parameters of 160 adolescents conceived after ART were compared with those of 1,351 adolescents who were spontaneously conceived at 14 and 17 years of age (66). All data were adjusted for multiple births, primiparity, whether the primary caregiver was a smoker, family financial problems in the preceding year, parental socioeconomic status, and maternal and paternal age at conception. They completed the Child Behaviour Checklist and the adolescent Youth Self-Report to assess internalising (withdrawn behaviour, somatic complaints, anxiety, and depression), externalising (delinquent and aggressive behaviour), and total behaviour. They also completed the “Beck Depression Inventory for Youth,” and their parents reported doctor attendance for the diagnosed conditions of anxiety, behavioural problems, attention problems, and depression. At both 14 and 17 years of age, ART-conceived adolescents demonstrated less externalising behaviour, which was concordant with their parents’ reports. At both 14 and 17 years of age, there were no differences in internalising behaviour between the two groups. However, at 14 years of age ART offspring had a higher incidence of clinical depression (12.6% vs. 8.5%), although there were no differences subsequently detected at 17 years of age. On further analysis of data within the Raine Study participants, this confirmed an association of adolescent depression with parental subfertility, as those adolescents born in the Raine cohort to subfertile (non-ART) parents showed higher rates of clinical depression than those born to fertile parents at 14 years of age (13.7% vs. 6.9%), raising the possibility that any differences may be environmental and not related to ART *per se* (66).

### Quality of life

Perhaps the best measure of “overall health” is quality of life, and it is very reassuring to see a recent report on the long-term follow-up of young adults born from IVF in Melbourne. The group undertook a quality of life assessment using the World Health Organization Quality of Life—Brief assessment. This long-term follow-up study reported that 193 ART-conceived young adults recorded higher quality of life scores than their 86 naturally conceived counterparts, particularly in their social relationships and environment domains (67).

## Conclusion

Evidence about the long-term health of children conceived through ART appears reassuring. However, studies in this field come with many limitations. Low numbers of participants is one of the major limitations, which often makes subgroup analyses for diverse types of ART or diverse types of infertility not feasible. Furthermore, studies are often limited by short follow-up periods because of the difficulty and costs involved in longitudinal study designs. Meta-analyses in this field are often restricted by heterogeneity because of differences in methodologies and definitions of various aspects of ART in different studies. In addition, the rapid changes over time in ART limit the generalisability and significance of long-term findings.

It is important that well-designed studies investigating the long-term health outcomes of ART-conceived offspring are undertaken. These studies should be large enough to control for the many different aspects of the ART procedure that are undertaken. Furthermore, in the follow-up assessments, as challenging as they may be, attempts should be made to study potential health influences related to parenting, early life environmental exposures, and social relationships during childhood and adolescence. Some of the observed differences noted, with respect to allergies, hay fever, mental health, and hospital attendance may be attenuated by some of these influences.

Despite these statements, the conclusions from the work from the childhood hospitalisation data from the United Kingdom, the long-term follow-up and quality of life study from the Melbourne research group, and the data published from the Western Australian Growing Up Healthy Study should go a long way to help reassure current and prospective parents who may ultimately require ART to conceive.
